# Steroidopathies and hormonal imbalance in children and adolescents with autism spectrum disorder

**DOI:** 10.1002/jcv2.70070

**Published:** 2025-11-20

**Authors:** Concetta de Giambattista, Patrizia Ventura, Samuele Cortese, Paolo Trerotoli, Alessandra Di Gioia, Lucia Margari

**Affiliations:** ^1^ Child Neuropsychiatric Unit DiMePRe‐J‐Department of Precision and Rigenerative Medicine‐Jonic Area University of Bari Aldo Moro Bari Italy; ^2^ Developmental EPI (Evidence Synthesis, Prediction, Implementation) Lab Centre for Innovation in Mental Health School of Psychology Faculty of Environmental and Life Sciences University of Southampton Southampton UK; ^3^ Clinical and Experimental Sciences (CNS and Psychiatry) Faculty of Medicine University of Southampton Southampton UK; ^4^ Hampshire and Isle of Wight Healthcare NHS Foundation Trust Southampton UK; ^5^ Hassenfeld Children's Hospital at NYU Langone New York University Child Study Center New York City New York USA; ^6^ DiMePRe‐J‐Department of Precision and Rigenerative Medicine‐Jonic Area University of Bari “Aldo Moro” Bari Italy; ^7^ Department Interdisciplinary of Medicine Medical Statistic University of Bari Aldo Moro Bari Italy

**Keywords:** autism spectrum disorder, neurodevelopment, steroid hormones, steroidopathies, testosterone

## Abstract

**Background:**

Autism Spectrum Disorder (ASD) is a complex neurodevelopmental condition with a multifactorial etiology, many aspects of which remain unclear. Emerging evidence suggests a potential association between ASD and clinical manifestations resulting from hormonal imbalances, henceforth named “steroidopathies.” The present study aims to investigate the association between ASD, steroidopathies and altered steroid hormone levels.

**Methods:**

A cohort of 100 individuals aged 9–18 years was examined, comprising 50 autistic individuals (25 males, 25 females) compared to 50 individuals diagnosed with other neuropsychiatric disorders (20 males, 30 females). Data were collected on steroidopathies (including hirsutism, severe acne, irregular menstrual cycles, polycystic ovary syndrome, endometriosis, pubertal disorders) in participants and their first‐degree relatives, as well as on maternal pregnancy and delivery complications. Blood samples were analyzed to assess hormonal profiles, including testosterone, estradiol, and vitamin D levels. A multivariate logistic regression analysis was conducted, with ASD status as the dependent variable and hormone levels, steroidopathies and other pregnancy‐related conditions as independent variables. Sex and age were included as covariates.

**Results:**

The results indicate a higher prevalence of steroidopathies among relatives of individuals with ASD compared to those with other neuropsychiatric disorders, with statistically significant differences in the frequency of thyroid pathologies (OR = 2.66, 95% CI: 1.05–6.76, *p* = 0.0394) and a borderline effect for maternal hormonal treatment during pregnancy (OR = 3.17, 95% CI: 0.99–10.08, *p* = 0.0501). Serum testosterone levels were significantly higher in autistic individuals compared to controls (median 0.50 vs. 0.275 ng/mL, *p* = 0.0057).

**Conclusion:**

These findings support the hypothesis of an association between steroidopathies and altered steroid hormone levels with ASD, suggesting that steroid hormone profiles could potentially serve as early biomarkers in future research. Furthermore, consideration of maternal endocrine health and prenatal exposures may contribute to a better understanding of ASD risk.

## Key points

**What's known?**

- Emerging evidence suggests a link between steroid hormone dysregulation and Autism Spectrum Disorder (ASD).
- Elevated prenatal androgens, such as testosterone, have been observed in individuals later diagnosed with ASD.
- Steroid‐related conditions like polycystic ovary syndrome (PCOS) appear more frequently in mothers or female relatives of individuals with ASD.

**What's new?**

- The present study investigates the relationship between ASD, steroidopathies, and steroid hormone profiles in a well‐characterized, homogeneous cohort balanced for age and sex.
- By including first‐degree relatives, it assesses potential familial hormonal patterns.
- To determine whether these hormonal alterations are specific to ASD, the study compares the ASD group with a homogeneous group of individuals diagnosed with other neuropsychiatric conditions.
- The inclusion of a balanced male/female ratio and first‐degree relatives in the sample represents a methodological strength, as autistic females are often underrepresented and few studies investigate familial patterns, which limits insights into potential hereditary contributions to ASD.

**What's relevant?**

- This study provides evidence supporting a potential link between hormonal imbalances and ASD, emphasizing the need for further investigation into steroid hormone roles in neurodevelopmental pathways.
- The findings identify potential early biomarkers to support diagnosis and preventive strategies.
- Results emphasize the importance of improving prenatal care protocols and integrating hormonal profiling into ASD diagnosis and management frameworks.

## BACKGROUND

Autism Spectrum Disorder is a lifelong neurodevelopmental condition whose prevalence has significantly increased over the past few decades (Maenner et al., 2023). It is characterized by difficulties in initiating and sustaining reciprocal interaction and communication, alongside restrictive, repetitive, and inflexible patterns of behaviors, interests, or activities. Diagnosis requires that these impairments be evident from early development and significantly affect daily functioning. However, symptoms may only become fully apparent as social demands and requirements exceed the individual's capacities or may be masked by learned strategies in later life (American Psychiatric Association, 2022).

The etiology of ASD is complex and multifactorial, stemming from a tangled interplay of genetic as well as epigenetic and environmental factors (Fu et al., 2022; Pugsley et al., 2022; Yap et al., 2023). Among these factors, the role of steroid hormones and associated clinical manifestations (referred to as steroidopathies) is gaining attention (Gillberg et al., 2017). Studies have reported that fetal testosterone levels inversely correlate with visual attachment, language development, social skills and Empathy Quotient of the mother and infant (Auyeung et al., 2010; Qiao et al., 2025); and that postnatal testosterone levels (total serum concentration, its free fraction, and related precursor) are elevated in individuals with autistic traits and children with ASD (Artık et al., 2023; He et al., 2023; Wan et al., 2024). Additionally, disruptions in estrogen receptor signaling (Hwang et al., 2021) and in cholesterol metabolism (Puljko et al., 2025) as well as dysregulation of the hypothalamic‐pituitary‐adrenal (HPA) axis (including altered circadian rhythms, abnormal cortisol stress responses, and low plasma vitamin D levels), have been observed in ASD individuals (Wang et al., 2022; Ward et al., 2023). Furthermore, conditions such as PCOS, hirsutism, obesity, menstrual irregularities, severe acne, and an elevated risk of reproductive system cancers have been noted in both ASD females and mothers of ASD individuals and symptoms of hyperandrogenism were associated with autistic traits (Gasser et al., 2020; Jiang et al., 2022; Ward et al., 2023).

## OBJECTIVE

Emerging evidence suggests a link between steroid hormone dysregulation and ASD, yet current research is hindered by several methodological limitations. Female participants are often underrepresented, and few studies include first‐degree relatives, limiting insight into potential familial patterns. To address these gaps, the present study investigates the relationship between ASD, steroidopathies, and steroid hormone profiles in a well‐characterized, homogeneous cohort balanced for age and sex. By including first‐degree relatives, the study seeks to clarify familial contributions to hormonal dysregulation. Moreover, to determine whether these hormonal alterations are specific to ASD, the study compares the ASD group with a homogeneous group of individuals diagnosed with other neuropsychiatric conditions. Ultimately, this research seeks to advance the understanding of ASD pathophysiology and identify potential early biomarkers to support diagnosis and preventive strategies.

Based on these objectives and prior evidence, we hypothesized that (i) first‐degree relatives of individuals with ASD would present a higher occurrence of endocrine disorders, (ii) individuals with ASD would show an increased prevalence of steroidopathies compared to individuals with other neuropsychiatric conditions, and (iii) individuals with ASD would display altered hormonal profiles, particularly elevated serum testosterone levels. Furthermore, given prior evidence of sex‐specific effects, we anticipated differences in hormonal alterations between males and females with ASD.

## METHODS

### Participants and procedures

In an attempt to create homogeneous groups, we consecutively recruited individuals referred to the Neuropsychiatry Unit of the Azienda Ospedaliero‐Universitaria Policlinico di Bari between January and October 2024. Inclusion criteria were an age range of 9–18 years and a diagnosis of neuropsychiatric disorders, including ASD, or other neurodevelopmental and psychiatric conditions such as attention deficit and hyperactivity disorder (ADHD), anxiety and depression, and conduct disorder. The Autism Group (AG) was recruited consecutively, including all individuals with an ASD diagnosis who met the inclusion criteria, until a total of 50 participants was reached. Recruitment ensured an equal sex distribution, with 25 males and 25 females enrolled in the order of their referral. The Neuropsychiatric Group (NPG) was then composed of the first 50 consecutive inpatients who met the inclusion criteria, had no ASD diagnosis, and concurrently matched the AG participants by age and sex. This procedure ensured demographic comparability between the two groups while preserving the consecutive recruitment within each group.

Information on perinatal history, including mode of delivery (spontaneous vaginal delivery, elective caesarean section, or medically indicated caesarean section), was systematically collected for all participants. At the time of recruitment, information on current medication use was collected. Individuals receiving hormonal therapy were excluded from the study. Furthermore, in the analyzed sample, no statistically significant differences were observed between the AG and NPG groups with respect to the use of other medications.

Detailed information about the purpose and the method of the study was provided to the participants and their families. Besides, written informed consent from parents was obtained before enrollment.

All participants were diagnosed through a complete neuropsychiatric evaluation by Neuropsychiatrist specialists belonging to the research team, employing standard assessment (including Child Behavior Checklist, CBCL, and Youth Self Report Form, YSR) supplemented by Baron‐Cohen (BC) questionnaires (Baron‐Cohen et al., 2001, 2003; Baron‐Cohen & Wheelwright, 2004) and the semi‐structured questionnaire inspired by Testosterone‐related Medical Questionnaire (Ingudomnukul et al., 2007) (Supporting Information S1: Appendix S1), from which we extracted the items most pertinent to our research objectives.

The study was approved by the Ethics Committee of the Azienda Ospedaliero‐Universitaria Consorziale Policlinico di Bari (study code: NEUROSVI‐2022, protocol number: 0015608). Informed consent was obtained from the parents of all participants.

### Data collection on familial and female participants' steroidopathies

Data on steroidopathies were collected through anamnestic interviews, supported by the semi‐structured questionnaire adapted from the Testosterone‐Related Medical Questionnaire.

Participants were asked whether any first‐degree relatives had ever experienced hormonal or gynecological issues (e.g., PCOS, endometriosis, precocious or delayed puberty, breast, ovarian, uterine or prostate cancer, metabolic diseases such as gestational diabetes). Maternal medical history was also recorded, including the use of hormonal treatments, diabetes, preeclampsia, thyroid disorders, stress, smoking, medication use, and assisted reproductive technologies during pregnancy, as well as delivery complications. Data were also collected on maternal and paternal ages and breastfeeding duration. Moreover, females were asked whether they had hirsutism, severe acne, irregular menstrual cycles, painful periods, or excessive menstrual bleeding.

### Hormonal and metabolic blood tests

Hormonal and Metabolic Blood Tests: Blood samples were collected from participants after fasting between 08:00 and 10:00 AM. The following hormone levels were measured: estradiol, prolactin, testosterone, folate, insulin, cortisol, and vitamin D. Electrochemiluminescence and chemiluminescence methods were used for the analysis.

### Statistical analyses

Quantitative variables (age, hormone levels, tests specific for autism, and tests specific for other co‐occurring conditions) were summarized as mean and standard deviation or median and interquartile ranges after assessing normality using the Shapiro–Wilk test. Independent group comparisons were conducted using *t*‐tests (parametric) or Wilcoxon tests (nonparametric); Hedges' effect size was determined in both cases to evaluate the magnitude of the difference. Association between variables was assessed using Spearman's rank coefficient, as many variables did not sufficiently approximate the Gaussian distribution. Qualitative characteristics (sex, presence/absence of other diagnoses) were summarized as counts and percentages, and comparisons between independent groups were made using a chi‐square test or Fisher's Exact test, as appropriate. To evaluate the magnitude of the effect, Cramer's effect size was determined. For quantitative variables that showed significant differences between the AG and NPG, an analysis was also carried out to determine whether it was possible to associate the outcome of the characteristic with the presence of ASD. In the case of qualitative features, for this purpose, logistic regression and ROC curve analysis were used, with determination of the area under the curve (AUC) and associated confidence interval. To identify the characteristics that best predicted the risk of ASD, a logistic regression analysis was performed, with ASD status as the dependent variable. First, a univariable analysis was conducted including all variables as independent variables; subsequently, a multivariable model was analyzed, with independent variables chosen among those that were statistically significant in the univariable analysis (these were testosterone level, medication use during pregnancy, and presence of thyroid disease). Sex and age were included as covariates. The analyses were conducted with SAS 9.4 personal computer software. A *p*‐value of < 0.05 was considered the threshold for defining statistical significance.

## FINDINGS

The demographic characteristics (age and sex; Table 1) did not significantly differ between the AG and the NPG. Similarly, scores on the BC questionnaires (autistic quotient, AQ; empathy quotient, EQ; systemizing quotient, SQ; as well as on the Child Behavior Checklist (CBCL) and the Youth Self Report (YSR; Table 1)), showed no significant differences between groups, with the exception of the CBCL subscales for Oppositional Defiant Disorder and Conduct Disorder, which were significantly higher in the NPG.

**TABLE 1:** Demographic characteristics and scores obtained by the two groups, AG and NPG, on the AQ, EQ, SQ, CBCL and YSR questionnaires.

	AG		NPG				
	Mean	IQR	Mean	IQR	*p*‐value	Hedge's effect size	Test statistic *Z*
Age	13	12.00–15.00	13	11.00–16.00	0.5697	0.0569	−0.569
	*N*	Percentage	*N*	Percentage		Cramer's *V* effect	Test chi‐square
M	25	50.00%	20	40.00%	0.3173	0.1	1.00
F	25	50.00%	30	60.00%			

	Mean	IQR	Mean	IQR	*p*‐value	Hedge's effect size	Test statistic *Z*
AQ	33.00	17.95	29.76	21.11	0.5145	19.6951	0.656
EQ	29.83	12.65	34.79	9.82	0.0859	11.2545	−1.745
SQ	42.10	17.80	38.97	16.94	0.4805	17.3466	0.71

	Mean	IQR	Mean	IQR	*p*‐value	Hedge's effect size	Test statistic *Z*
Affective CBCL	70	65.25–75.00	70	63.00–75.00	0.7895	0.03348	0.272
Anxiety CBCL	70	63.50–72.25	70	60.00–72.25	0.9690	0.0048	0.0388
Somatic CBCL	59	55.50–64.50	59	50.00–62.50	0.5267	0.0779	0.633
ADHD prob. CBCL	59	54.50–67.25	60	56.00–70.00	0.2494	0.1418	1.152
Oppositional defiant CBCL	52	52.00–62.25	62	56.00–71.00	0.0010	0.4047	3.288
Conduct CBCL	55	50.00–60.25	65	51.00–71.00	0.0069	0.3350	2.701
Affective YSR	70	62.00–75.75	63	56.00–72.00	0.2655	0.1662	−1.115
Anxiety YSR	68	56.75–73.75	68	55.00–70.00	0.4390	0.1157	−0.776
Somatic YSR	56	51.25–68.00	60	52.00–66.00	0.4456	0.1140	−0.765
ADHD YSR	57	52.50–68.00	61.50	51.00–68.00	0.9274	0.0136	−0.0913
Oppositional defiant YSR	56	52.00–61.00	60	55.00–65.00	0.1512	0.21467	−1.44
Conduct YSR	55	50.25–61.25	57	50.00–73.00	0.4343	0.1170	−0.785

Abbreviations: AG, Autism Group; AQ, autistic quotient; CBCL, Child Behavior Checklist; EQ, empathizing quotient; IQR, InterQuartile Range; NPG, NeuroPsychiatric Group; SD, standard deviation; SQ, systematizing quotient; YSR, Youth Self Report.

No significant differences were found between AG and NPG with respect to mode of delivery. The frequency distribution of spontaneous vaginal delivery (40.4% vs. 55.8%), scheduled caesarean section (25.5% vs. 20.9%), and emergency caesarean section (23.4% vs. 14.0%) was comparable across the two groups and was further confirmed by logistic regression, where the association between type of delivery and AG/NPG status was not statistically significant (Type III analysis of effects, Wald chi‐square = 2.38, df = 3, *p* = 0.4965; Supporting Information S1: Table S2). Similarly, no statistically significant differences were observed between the two groups regarding the use of medications at the time of recruitment.

### Familial steroidopathies

In terms of familial steroidopathies, no significant differences were found in the prevalence of conditions like PCOS or endometriosis between the AG and NPG. Detailed data on familial, maternal, and paternal steroidopathies in the AG and Neuropsychiatric Group (NPG) are presented in Supporting Information S1: Table S1.

However, there was a significantly higher prevalence of thyroid pathologies in the ASD group compared to NPG families (57.4% vs. 31.1%, Cramer's *V* = 0.265; Fisher's Exact test, *p* = 0.0115). Furthermore, the univariate analysis showed that thyroid pathologies in the family were associated with a statistically significant risk of ASD (OR = 2.98, 95% CI: 1.27–7.04, *p* = 0.0122; Supporting Information S1: Table S2). Maternal hormonal treatment during pregnancy (with progesterone being the most frequently reported medication among those cited by the interviewed caregivers) was not associated with a statistically significant risk of belonging to the AG family (OR = 1.05, 95% CI: 0.46–2.41, *p* = 0.9037; Supporting Information S1: Table S2).

### Female ASD and NP participants' steroidopathies

Although certain steroidopathies (hirsutism, severe acne, and irregular menstrual cycles) appeared more frequently in the AG compared to the NPG, these differences did not reach statistical significance (Table 2).

**TABLE 2:** Female ASD and NP participants' steroidopathies.

	AG		NPG			
	Present/total valid answer (*n* = 24)	%	Present/total valid answer (*n* = 26)	%	*p*‐value	Cramer's *V* effect
Hirsutism	7	29.20	4	15.40	0.3138	0.166
Severe acne	4	16.70	4	15.40	1.0000	0.017
Irregular menstrual cycle	6	25.00	6	23.10	1.0000	0.022
Painful menstrual cycle	3	12.50	4	15.40	1.0000	0.041
Excessive bleeding during menstrual cycle	3	12.50	2	7.70	0.6613	0.08

Abbreviations: AG, Autism Group; ASD, autism spectrum disorder; NPG, NeuroPsychiatric Group.

### Hormonal and metabolic blood values

Hormonal blood tests revealed significantly higher serum testosterone levels in the AG compared to the NPG (0.50 ng/mL vs. 0.275 ng/mL, Hedge's effect size = 0.28, *p* = 0.0057), with no significant differences in estradiol, prolactin, or vitamin D levels (Table 3).

**TABLE 3:** Hormonal and metabolic values in AG and NPG.

	AG		NPG				
	Median	IQR	Median	IQR	*p*‐value	Hedge's effect size	Test statistic *Z*
Estradiol (pg/mL)	42.25	20.10–91.80	42.30	7.20–86.20	0.3697	0.09	0.897
Prolactin (pg/mL)	10.50	6.15–18.92	11.55	7.80–16.20	0.6119	0.05	0.507
Testosterone (ng/mL)	0.50	0.26–4.98	0.275	0.14–0.54	0.0057	0.28	2.767
Folates (ng/mL)	4.15	3.30–5.30	3.90	2.95–5.17	0.3970	0.09	0.847
Insulin (microUI/mL)	9.85	6.90–13.10	11.70	7.60–16.00	0.2290	0.12	1.203
Cortisol (ug/L)	89.00	67.50–142.50	87.00	61.750–140.25	0.5916	0.05	0.536
Vitamin D (ng/mL)	21.00	16.00–26.25	20.50	16.00–27.00	0.7001	0.04	0.385

Abbreviations: AG, Autism Group; IQR, InterQuartile Range; NPG, NeuroPsychiatric Group.

Variables that reached statistical significance in the univariate analysis (Supporting Information S1: Table S2) were subsequently included in the multivariate model (Table 4). Subsequently, results of the multivariate logistic regression analysis have shown that thyroid pathology (OR = 2.98; CI 95% 1.27–7.035; *p* = 0.0122), medication use during pregnancy (OR = 3.56 CI 95%1.25–10.11; *p* = 0.017), serum testosterone (OR = 1.45; CI 95% 1.06–1.98; *p* = 0.0208) are independent risk factors for ASD regardless of sex, age in children (Table 4; Supporting Information S1: Table S2).

**TABLE 4:** Results of the multivariable logistic regression analysis, and corresponding 95% confidence interval.

Variable		OR	95% CI		*p*‐value
Age	For each 1 year increase	0.942	0.765	1.16	0.5731
Sex	F versus M	2.075	0.548	7.861	0.2828
Thyroid disease	Yes versus No	2.662	1.049	6.759	0.0394
Medication during pregnancy	Yes versus No	3.174	0.999	10.079	0.0501
Testosterone	For each 1 unit increase	1.45	1.058	1.988	0.0208

Abbreviations: CI, confidence interval; OR, odds ratio.

**FIGURE 1:**
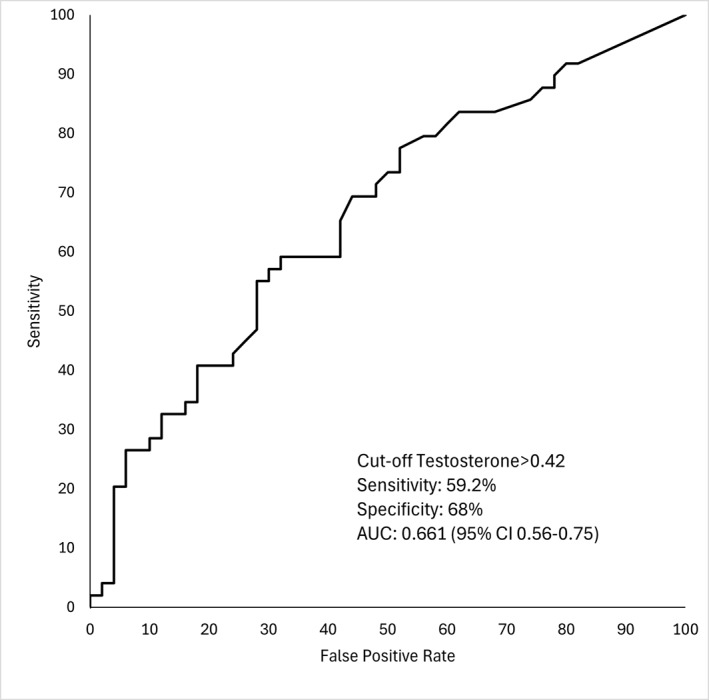
ROC curves of testosterone.

To have a suggestion of a discriminant threshold for testosterone that could determine an increased risk of ASD, a ROC analysis was conducted (Figure 1), and it has resulted with a mild AUC 0.66 (95% CI 0.56–0.75), and the risk for ASD increases for a testosterone level > 0.42 ng/mL.

Association analysis between scores and hormone levels revealed a statistically significant correlation only between testosterone and SQ scores in males, in both the AG (*r* = 0.7, *p* = 0.0019) and the NPG (*r* = 0.7, *p* = 0.0123; Supporting Information S1: Table S3).

## DISCUSSION

This study aimed to investigate the association between ASD, steroidopathies, and steroid hormone profiles, with a particular focus on familial endocrine conditions and maternal factors. Our findings indicate that steroid hormone dysregulation in ASD persists after controlling for sex distribution, familial history, and comparison with other neuropsychiatric conditions. Specifically, elevated serum testosterone, increased prevalence of familial thyroid pathology, and maternal hormonal treatment during pregnancy emerged as independent risk factors for ASD. These results provide new evidence linking hormonal alterations with ASD risk and pathophysiology and suggest potential pathways for identifying early biomarkers.

Steroid hormones have been investigated in recent years for their potential contribution to the etiopathogenesis of ASD, from BC's studies on the Extreme Male Brain (Baron‐Cohen, 2002), to Gillberg's hypotheses regarding the impact of cholesterol metabolism alterations on neurological development (Gillberg et al., 2017), to Pohl's research on the presence of steroidopathies in autistic females (Pohl et al., 2024), and Lai's analysis of the influence of prenatal androgens on the social brain (Lombardo et al., 2020). These hormones are essential for neurodevelopment, influencing neural proliferation, neurotransmission, oxidative stress regulation, and immune function. They may also affect cerebrovascular development, act as epigenetic fetal programming factors shaping the early brain environment and interact with thyroid and glucocorticoid hormones—via the placenta as a mediator—thereby influencing brain development (Banik et al., 2017; Collignon et al., 2024; Ward et al., 2023).

This study supports the notion that steroid hormonal abnormalities, particularly elevated serum testosterone levels, may represent risk factors for ASD.

### Familial steroidopathies

Previous studies, comparing ASD individuals with neurotypical children, have indicated that maternal conditions characterized by a hyper‐androgenic status, such as PCOS, are linked to a higher probability of offspring being diagnosed with autism (Cesta et al., 2020; Katsigianni et al., 2019; Kosidou et al., 2016). Kosidou et al. (2016), in a large population‐based study conducted in Sweden, reported that maternal PCOS increased the odds of ASD in offspring by 59%, with no differences observed between sexes (Kosidou et al., 2016). Katsigianni et al. (2019), through a systematic review and meta‐analysis, concluded that women with PCOS show increased odds of having a child with ASD, based on a large sample and high‐quality studies (Katsigianni et al., 2019). Expanding on these findings, Cesta et al. (2020) analyzed data from over one million births in the Swedish national registry and confirmed that children ‐especially females‐of mothers with PCOS may have a higher likelihood of developing neuropsychiatric disorders, particularly ASD, ADHD, and Tourette Syndrome (Cesta et al., 2020).

Although the difference in the prevalence of familial steroidopathies between individuals with ASD and those with other neuropsychiatric disorders was not statistically significant, this study found a significantly higher prevalence of familial thyroid pathologies in the ASD group, independent of sex and age. This finding confirms previous studies suggesting that thyroid dysfunction could be a risk factor for ASD. Thyroid hormones are essential for neuronal differentiation, synaptogenesis, and myelination in the fetal brain. Variations in maternal thyroid hormone levels (also associated with thyroid autoimmunity) particularly during the first trimester, may exert a significant impact on fetal brain development, increasing the risk for ASD and other neurodevelopmental disorders (Rotem et al., 2020; Ward et al., 2023).

Regarding maternal medication use, our statistical analysis indicates that the use of at least one medication during pregnancy is a borderline significant risk factor. The most commonly used medications by our participants' mothers during pregnancy, apart from those for thyroid conditions, are anti‐abortive drugs, primarily progesterone. Our results are consistent with recent scientific literature, including both animal models and population‐based case‐control studies (Davidovitch et al., 2018; Zou et al., 2017), which have shown that progesterone exposure during fetal life may increase the risk of ASD, potentially through epigenetic modification. Davidovitch et al. (2018) found that while infertility treatments themselves were not directly linked to a significant increase in ASD risk, the use of progesterone during early pregnancy may contribute to a higher likelihood of ASD development (Davidovitch et al., 2018). Similarly, Zou (2017) investigated the relationship between prenatal exposure to synthetic progestins, the downregulation of estrogen receptors, and the emergence of cognitive deficits and autism‐like behaviors in both animal models and national registry datasets (Zou et al., 2017).

### Female ASD and NP participants' steroidopathies

In the female AG, a high prevalence of steroidopathy‐related symptoms was observed, including hirsutism (29.2%), acne (16.7%), and menstrual irregularities (25.0%). Although these rates were not statistically different from those observed in the NPG, they represent clinically relevant findings within the sample. It is also worth noting that the study population ranged from 9 to 18 years of age, which may precede the typical age of onset for some of these conditions, potentially leading to an underestimation of their actual prevalence. To better contextualize these findings, it is important to compare them with prevalence estimates from the general population, where hirsutism affects approximately 4%–10% of women worldwide (Spritzer et al., 2022), acne affects approximately 9.4% (Tan & Bhate, 2015), and menstrual irregularities range from 14% to 25% (American College of Obstetricians and Gynecol ogists (ACOG) Committee Opinion No. 760, 2018). The presence of alterations in steroid hormones—particularly in females with ASD‐ and the related expression of clinical conditions known as steroidopathies‐have been extensively discussed in recent literature (Ward et al., 2023). Ingudomnukul, Pohl, and Ward (2007, 2014, 2023) have explored the increased prevalence of testosterone related disorders and steroidopathic symptoms in women with ASD compared to neurotypical controls (Ingudomnukul et al., 2007; Pohl et al., 2024; Ward et al., 2023).

### Hormonal blood values

From BC's early studies to more recent research, attention has increasingly focused on the possibility of measuring these steroid hormonal alterations by assessing prenatal and postnatal steroid hormone levels in amniotic fluid and in other bodily fluids (saliva, urine, blood) (Artık et al., 2023; Auyeung et al., 2010; He et al., 2023; Katsigianni et al., 2019; Qiao et al., 2025; Wan et al., 2024). Baron‐Cohen et al. analyzed amniotic fluid samples, searching for androgens in 2015 (Baron‐Cohen et al., 2015) and estrogens in 2020 (Baron‐Cohen et al., 2020), finding an association between hormonal levels and likelihood of autism. Gasser et al. (2020) found, in a small group of autistic female adolescents, elevated androgen levels compared to neurotypical controls (Gasser et al., 2020). Artik et al. (2023) found that serum levels of testosterone in prepubertal drug‐naive boys with ADHD were associated with more pronounced autistic traits (Artık et al., 2023). He et al. (2023) investigated salivary steroid hormones, variations in children and adolescents with ASD versus neurotypical controls, finding that subjects with ASD had significantly higher salivary testosterone precursors levels (dehydroepiandrosterone and pregnenolone), than their neurotypical peers (He et al., 2023). Wang et al. (2024) provided a systematic review and meta‐analysis on androgen levels in blood, urine, saliva finding significantly higher serum free testosterone levels, in individuals with ASD compared to control groups and total testosterone and DHEA in urine (Wan et al., 2024).

In the present study, we analyzed serum hormone levels in children and adolescents with ASD and other neuropsychiatric conditions, finding that serum testosterone is an independent risk factor in autistic children regardless of sex or age. Moreover, our results showed that serum testosterone levels are associated with hyper‐systemizing traits, measured with the SQ, exclusively in males ‐both in those diagnosed with autism and those diagnosed with other neuropsychiatric disorders (Supporting Information S1: Table S2). These results are consistent with previous literature, which stems from BC's Extreme Male Brain theory demonstrating that elevated testosterone levels are associated with higher scores of autistic traits measured by BC's scales (Baron‐Cohen, 2002; Baron‐Cohen et al., 2020). Although the differences between these scores in ASD and non‐ASD groups were not statistically significant, our results suggest that the role of testosterone may be significant for autistic traits across diagnostic categories, rather than solely in cases of confirmed autism diagnosis ‐ as a trans‐nosographic feature. In addition, when comparing the ASD group with the NP group, a higher number of individuals with Oppositional Defiant Disorder—a condition notably associated with elevated testosterone levels (Shenk et al., 2012)—was observed in the NP group. However, in our ASD group, testosterone values were still higher, suggesting the significant role and the specificity of testosterone levels associated with autism, regardless of the presence of other disorders.

### Limitations

The main limitation of this study is the absence of a control group of healthy subjects. Additionally, the sample size may not be sufficient to allow for generalization of the findings to the broader population. Another limitation concerns potential bias due to the retrospective nature of the study, which required reliance on available clinical data. Further studies with larger sample sizes and an appropriate healthy control group are needed to confirm and expand upon these results.

## CONCLUSION

This study provides evidence of an association between ASD and altered steroid hormone levels, particularly elevated serum testosterone. Familial steroidopathies, especially thyroid pathologies, were more prevalent in families of individuals with ASD. These findings suggest that steroidopathies and hormonal imbalances could play a role in the etiopathogenesis of ASD.

## CLINICAL IMPLICATIONS

The association between ASD and elevated testosterone levels suggests a potential biomarker for early identification or risk stratification. Furthermore, the role of maternal hormonal imbalance and drug exposure during pregnancy as potential risk factors highlights the need for careful prenatal monitoring and risk evaluation.

## AUTHOR CONTRIBUTIONS

**Concetta de Giambattista**: Conceptualization; methodology; writing—original draft; data curation; investigation. **Patrizia Ventura**: Methodology; investigation; writing—review and editing. **Samuele Cortese**: Supervision; writing—review and editing; conceptualization. **Paolo Trerotoli**: Methodology; validation; writing—review and editing. **Alessandra Di Gioia**: Investigation; writing—review and editing. **Lucia Margari**: Conceptualization; methodology; writing—original draft; writing—review and editing; funding acquisition; investigation.

## CONFLICT OF INTEREST STATEMENT

Prof. S.C. has declared reimbursement for travel and accommodation expenses from the Association for Child and Adolescent Central Health (ACAMH) in relation to lectures delivered for ACAMH, the Canadian AADHD Alliance Resource, the British Association of Psychopharmacology, Healthcare Convention and CCM Group team for educational activity on ADHD, and has received honoraria from Medice. The remaining authors have declared that they have no competing or potential conflicts of interest.

## ETHICAL CONSIDERATIONS

The study was conducted in accordance with the Declaration of Helsinki. Informed consent was obtained from all participants and/or their parents or legal guardians. The research protocol was approved by the Ethics Committee of the Azienda Ospedaliera‐Universitaria Consorziale Policlinico di Bari (Italy) on 16 February 2023 (approval number 7573).

## Supporting information

Supporting Information S1

## Data Availability

The data that support the findings of this study are available on request from the corresponding author. The data are not publicly available due to privacy or ethical restrictions.

## References

[jcv270070-bib-0001] American College of Obstetricians and Gynecologists (ACOG) Committee Opinion No. 760 . (2018). Dysmenorrhea and endometriosis in the adolescent. Obstetrics & Gynecology, 132(6), e249–e258. 10.1097/AOG.0000000000002978 30461694

[jcv270070-bib-0002] American Psychiatric Association . (2022). Diagnostic and statistical manual of mental disorders fifth ed., text rev (DSM‐5 TR). American Psychiatric Association.

[jcv270070-bib-0003] Artık, A. , Çengel Kültür, S. E. , Portakal, O. , & Karaboncuk, A. Y. (2023). The association between autistic traits and serum testosterone, oxytocin, and androstenedione levels in prepubertal male drug naive children with attention‐deficit/hyperactivity disorder. International Journal of Developmental Neuroscience, 83(1), 98–107. 10.1002/jdn.10241 36398591

[jcv270070-bib-0004] Auyeung, B. , Taylor, K. , Hackett, G. , & Baron‐Cohen, S. (2010). Foetal testosterone and autistic traits in 18 to 24‐month‐old children. Molecular Autism, 1(1), 11. 10.1186/2040-2392-1-11 20678186 PMC2916006

[jcv270070-bib-0005] Banik, A. , Kandilya, D. , Ramya, S. , Stünkel, W. , Chong, Y. S. , & Dheen, S. T. (2017). Maternal factors that induce epigenetic changes contribute to neurological disorders in offspring. Genes, 8(6), 150. 10.3390/genes8060150 28538662 PMC5485514

[jcv270070-bib-0006] Baron‐Cohen, S. (2002). The extreme male brain theory of autism. Trends in Cognitive Sciences, 6(6), 248–254. 10.1016/s1364-6613(02)01904-6 12039606

[jcv270070-bib-0007] Baron‐Cohen, S. , Auyeung, B. , Nørgaard‐Pedersen, B. , Hougaard, D. M. , Abdallah, M. W. , Melgaard, L. , Cohen, A. S. , Chakrabarti, B. , Ruta, L. , & Lombardo, M. V. (2015). Elevated fetal steroidogenic activity in autism. Molecular Psychiatry, 20(3), 369–376. 10.1038/mp.2014.48 24888361 PMC4184868

[jcv270070-bib-0008] Baron‐Cohen, S. , Richler, J. , Bisarya, D. , Gurunathan, N. , & Wheelwright, S. (2003). The systemizing quotient: An investigation of adults with Asperger syndrome or high‐functioning autism, and normal sex differences. Philosophical Transactions of the Royal Society of London B Biological Sciences, 358(1430), 361–374. 10.1098/rstb.2002.1206 12639333 PMC1693117

[jcv270070-bib-0009] Baron‐Cohen, S. , Tsompanidis, A. , Auyeung, B. , Nørgaard‐Pedersen, B. , Hougaard, D. M. , Abdallah, M. , Cohen, A. , & Pohl, A. (2020). Foetal oestrogens and autism. Molecular Psychiatry, 25(11), 2970–2978. 10.1038/s41380-019-0454-9 31358906 PMC7577840

[jcv270070-bib-0010] Baron‐Cohen, S. , & Wheelwright, S. (2004). The empathy quotient: An investigation of adults with Asperger syndrome or high functioning autism, and normal sex differences. Journal of autism and developmental disorders. Journal of Autism and Developmental Disorders, 34(2), 163–175. 10.1023/b:jadd.0000022607.19833.00 15162935

[jcv270070-bib-0011] Baron‐Cohen, S. , Wheelwright, S. , Skinner, R. , Martin, J. , & Clubley, E. (2001). The autism‐spectrum quotient (AQ): Evidence from Asperger syndrome/high‐functioning autism, males and females, scientists and mathematicians. Journal of Autism and Developmental Disorders, 31(1), 5–17. 10.1023/a:1005653411471 11439754

[jcv270070-bib-0012] Cesta, C. E. , Öberg, A. S. , Ibrahimson, A. , Yusuf, I. , Larsson, H. , Almqvist, C. , D'Onofrio, B. M. , Bulik, C. M. , Fernández de la Cruz, L. , Mataix‐Cols, D. , Landén, M. , & Rosenqvist, M. A. (2020). Maternal polycystic ovary syndrome and risk of neuropsychiatric disorders in offspring: Prenatal androgen exposure or genetic confounding? Psychological Medicine, 50(4), 616–624. 10.1017/S0033291719000424 30857571 PMC7093321

[jcv270070-bib-0013] Collignon, A. , Dion‐Albert, L. , Ménard, C. , & Coelho‐Santos, V. (2024). Sex, hormones and cerebrovascular function: From development to disorder. Fluids and Barriers of the CNS, 21(1), 2. 10.1186/s12987-023-00496-3 38178239 PMC10768274

[jcv270070-bib-0014] Davidovitch, M. , Chodick, G. , Shalev, V. , Eisenberg, V. H. , Dan, U. , Reichenberg, A. , Sandin, S. , & Levine, S. Z. (2018). Infertility treatments during pregnancy and the risk of autism spectrum disorder in the offspring. Progress in Neuro‐Psychopharmacology and Biological Psychiatry, 30(86), 175–179. 10.1016/j.pnpbp.2018.05.022 29864450

[jcv270070-bib-0015] Fu, J. M. , Satterstrom, F. K. , Peng, M. , Brand, H. , Collins, R. L. , Dong, S. , Wamsley, B. , Klei, L. , Wang, L. , Hao, S. P. , Stevens, C. R. , Cusick, C. , Babadi, M. , Banks, E. , Collins, B. , Dodge, S. , Gabriel, S. B. , Gauthier, L. , Lee, S. K , … Sanders, S. J. (2022). Rare coding variation provides insight into the genetic architecture and phenotypic context of autism. Nature Genetics, 54(9), 1320–1331. 10.1038/s41588-022-01104-0 35982160 PMC9653013

[jcv270070-bib-0016] Gasser, B. A. , Kurz, J. , Dick, B. , & Mohaupt, M. G. (2020). Are steroid hormones dysregulated in autistic girls? Diseases, 8(1), 6. 10.3390/diseases8010006 32183287 PMC7151154

[jcv270070-bib-0017] Gillberg, C. , Fernell, E. , Kočovská, E. , Minnis, H. , Bourgeron, T. , Thompson, L. , & Allely, C. S. (2017). The role of cholesterol metabolism and various steroid abnormalities in autism spectrum disorders: A hypothesis paper. Autism Research, 10(6), 1022–1044. 10.1002/aur.1777 28401679 PMC5485071

[jcv270070-bib-0018] He, Q. , Wang, Y. , Liu, Z. , Xia, J. , Yin, H. , Qiu, Z. , Wang, H. , Xu, W. , Xu, Z. , & Xie, J. (2023). Analysis of salivary steroid hormones in boys with autism spectrum disorder. BMC Psychiatry, 23(1), 105. 10.1186/s12888-023-04586-2 36788524 PMC9926760

[jcv270070-bib-0019] Hwang, W. J. , Lee, T. Y. , Kim, N. S. , & Kwon, J. S. (2021). The role of estrogen receptors and their signaling across psychiatric disorders. International Journal of Molecular Sciences, 22(1), 373. 10.3390/ijms22010373 PMC7794990 33396472

[jcv270070-bib-0020] Ingudomnukul, E. , Baron‐Cohen, S. , Wheelwright, S. , & Knickmeyer, R. (2007). Elevated rates of testosterone‐related disorders in women with autism spectrum conditions. Hormones and Behavior, 51(5), 597–604. 10.1016/j.yhbeh.2007.02.001 17462645

[jcv270070-bib-0021] Jiang, L. , Tian, L. , Yuan, J. , Xu, X. , Qu, F. , Zhang, R. , & Wang, J. (2022). Associations between sex hormone levels and autistic traits in infertile patients with polycystic ovary syndrome and their offspring. Frontiers in Endocrinology, 12, 789395. 10.3389/fendo.2021.789395 35173679 PMC8842647

[jcv270070-bib-0022] Katsigianni, M. , Karageorgiou, V. , Lambrinoudaki, I. , & Siristatidis, C. (2019). Maternal polycystic ovarian syndrome in autism spectrum disorder: A systematic review and meta‐analysis. Molecular Psychiatry, 24(12), 1787–1797. 10.1038/s41380-019-0398-0 30867561

[jcv270070-bib-0023] Kosidou, K. , Dalman, C. , Widman, L. , Arver, S. , Lee, B. K. , Magnusson, C. , & Gardner, R. M. (2016). Maternal polycystic ovary syndrome and the risk of autism spectrum disorders in the offspring: A population‐based nationwide study in Sweden. Molecular Psychiatry, 21(10), 1441–1448. 10.1038/mp.2015.183 26643539 PMC5030459

[jcv270070-bib-0024] Lombardo, M. V. , Auyeung, B. , Pramparo, T. , Quartier, A. , Courraud, J. , Holt, R. J. , Waldman, J. , Ruigrok, A. N. V. , Mooney, N. , Bethlehem, R. A. I. , Lai, M. C. , Kundu, P. , Bullmore, E. T. , Mandel, J. L. , Piton, A. , & Baron‐Cohen, S. (2020). Sex‐specific impact of prenatal androgens on social brain default mode subsystems. Molecular Psychiatry, 25(9), 2175–2188. 10.1038/s41380-018-0198-y 30104728 PMC7473837

[jcv270070-bib-0025] Maenner, M. J. , Warren, Z. , Williams, A. R. , Amoakohene, E. , Bakian, A. V. , Bilder, D. A. , Durkin, M. S. , Fitzgerald, R. T. , Furnier, S. M. , Hughes, M. M. , Ladd‐Acosta, C. M. , McArthur, D. , Pas, E. T. , Salinas, A. , Vehorn, A. , Williams, S. , Esler, A. , Grzybowski, A. , Hall‐Lande, J , … Washington, A. (2023). Prevalence and characteristics of autism spectrum disorder among children aged 8 years—Autism and developmental disabilities monitoring network, 11 sites, United States, 2020. MMWR. Surveillance Summaries, 72(2), 1–14. 10.15585/mmwr.ss7202a1 PMC10042614 36952288

[jcv270070-bib-0026] Pohl, A. , Cassidy, S. , Auyeung, B. , & Baron‐Cohen, S. (2024). Uncovering steroidopathy in women with autism: A latent class analysis. Molecular Autism, 5(1), 27. 10.1186/2040-2392-5-27 PMC4022124 24717046

[jcv270070-bib-0027] Pugsley, K. , Scherer, S. W. , Bellgrove, M. A. , & Hawi, Z. (2022). Environmental exposures associated with elevated risk for autism spectrum disorder may augment the burden of deleterious de novo mutations among probands. Molecular Psychiatry, 27(1), 710–730. 10.1038/s41380-021-01142-w 34002022 PMC8960415

[jcv270070-bib-0028] Puljko, B. , Štracak, M. , Kalanj‐Bognar, S. , Todorić Laidlaw, I. , & Mlinac‐Jerkovic, K. (2025). Gangliosides and cholesterol: Dual regulators of neuronal membrane framework in autism spectrum disorder. International Journal of Molecular Sciences, 26(3), 1322. 10.3390/ijms26031322 39941090 PMC11818915

[jcv270070-bib-0029] Qiao, D. , Mu, C. , Chen, H. , Wen, D. , Wang, Z. , Zhang, B. , Guo, F. , Wang, C. , Zhang, R. , Wang, C. , Cui, H. , & Li, S. (2025). Implications of prenatal exposure to hyperandrogen for hippocampal neurodevelopment and autism‐like behavior in offspring. Progress In Neuro‐Psychopharmacology & Biological Psychiatry, 10, 136, 111219. 10.1016/j.pnpbp.2024 39694316

[jcv270070-bib-0030] Rotem, R. S. , Chodick, G. , Shalev, V. , Davidovitch, M. , Koren, G. , Hauser, R. , Coull, B. A. , Seely, E. W. , Nguyen, V. T. , & Weisskopf, M. G. (2020). Maternal thyroid disorders and risk of autism spectrum disorder in Progeny. Epidemiology, 31(3), 409–417. 10.1097/EDE.0000000000001174 32251066

[jcv270070-bib-0031] Shenk, C. E. , Dorn, L. D. , Kolko, D. J. , Susman, E. J. , Noll, J. G. , & Bukstein, O. G. (2012). Predicting treatment response for oppositional defiant and conduct disorder using pre‐treatment adrenal and gonadal hormones. Journal of Child and Family Studies, 21(6), 973–981. 10.1007/s10826-011-9557-x 27429540 PMC4943761

[jcv270070-bib-0032] Spritzer, P. M. , Marchesan, L. B. , Santos, B. R. , & Fighera, T. M. (2022). Hirsutism, normal androgens and diagnosis of PCOS. Diagnostics, 12(8), 1922. 10.3390/diagnostics12081922 36010272 PMC9406611

[jcv270070-bib-0033] Tan, J. K. , & Bhate, K. (2015). A global perspective on the epidemiology of acne. British Journal of Dermatology, 172(Suppl 1), 3–12. 10.1111/bjd.13462 25597339

[jcv270070-bib-0034] Wang, J. , Huang, H. , Liu, C. , Zhang, Y. , Wang, W. , Zou, Z. , Yang, L. , He, X. , Wu, J. , Ma, J. , & Liu, Y. (2022). Research progress on the role of vitamin D in autism spectrum disorder. Frontiers in Behavioral Neuroscience, 10, 16, 859151. 10.3389/fnbeh.2022.859151 PMC9128593 35619598

[jcv270070-bib-0035] Wang, Z. , Zhang, B. , Mu, C. , Qiao, D. , Chen, H. , Zhao, Y. , Cui, H. , Zhang, R. , & Li, S. (2024). Androgen levels in autism spectrum disorders: A systematic review and meta‐analysis. Frontiers in Endocrinology, 15, 1371148. 10.3389/fendo.2024.1371148 38779452 PMC11109388

[jcv270070-bib-0036] Ward, J. H. , Weir, E. , Allison, C. , & Baron‐Cohen, S. (2023). Increased rates of chronic physical health conditions across all organ systems in autistic adolescents and adults. Molecular Autism, 14(1), 35. 10.1186/s13229-023-00565-2 37730651 PMC10510241

[jcv270070-bib-0037] Yap, C. X. , Henders, A. K. , Alvares, G. A. , Giles, C. , Huynh, K. , Nguyen, A. , Wallace, L. , McLaren, T. , Yang, Y. , Hernandez, L. M. , Gandal, M. J. , Hansell, N. K. , Cleary, D. , Grove, R. , Hafekost, C. , Harun, A. , Holdsworth, H. , Jellett, R. , Khan, F. , & Gratten, J. (2023). Interactions between the lipidome and genetic and environmental factors in autism. Nature Medicine, 29(4), 936–949. 10.1038/s41591-023-02271-1 PMC10115648 37076741

[jcv270070-bib-0038] Zou, Y. , Lu, Q. , Zheng, D. , Chu, Z. , Liu, Z. , Chen, H. , Ruan, Q. , Ge, X. , Zhang, Z. , Wang, X. , Lou, W. , Huang, Y. , Wang, Y. , Huang, X. , Liu, Z. , Xie, W. , Zhou, Y. , & Yao, P. (2017). Prenatal levonorgestrel exposure induces autism‐like behavior in offspring through ERβ suppression in the amygdala. Molecular Autism, 8(1), 46. 10.1186/s13229-017-0159-3 28824796 PMC5561609

