# Supplementary material for: Steroidopathies and hormonal imbalance in children and adolescents with autism spectrum disorder
Source: JCPP Adv. 2025 Nov 20;6(3):e70070. doi: 10.1002/jcv2.70070 (PMC13339666; doi:10.1002/jcv2.70070)
Supplement: Supplementary file 1 [file JCV2-6-e70070-s001.docx]

**STEROIDOPATHIES AND HORMONAL IMBALANCE IN CHILDREN AND ADOLESCENTS WITH AUTISM SPECTRUM DISORDER**

**Supporting Information**

**Appendix S1.** Semi-structured Questionnaire inspired by Testosterone-related Medical Questionnaire

1. Your child's name:
2. Your child’s age:
3. Indicate if your child is (natural/adoptive/in foster care):

For each question, please type Y for yes and N for no.

A. Have any of the minor’s first-degree relatives been diagnosed with any of the following?

# Y/N

1. Polycystic ovary syndrome (PCOS)
2. Endometriosis
3. Precocious or delayed puberty
4. Breast cancer
5. Ovarian cancer 6. Uterine cancer
7. Prostate cancer
8. Diabetes
7. Obesity
8. Thyroid pathologies
9. Other hormonal/gynecological conditions

B. Has the minor's mother ever had maternal hormonal treatments (including the contraceptive pill)?

# Y/N

C. Has the minor’s mother during pregnancy ever been diagnosed by a medical doctor with any of the following medical conditions? If yes, please specify.

# Y/N

1..Gestational diabetes

2. Preeclampsia
3. Maternal Thyroid pathologies
4. Threatened miscarriage
5. Intense emotional/physical stress
6. Smoke
7. Medication during pregnancy
8. If you have taken medication during pregnancy, please list them

D. Conception was:

# Y/N

1. Natural
2. With assisted reproduction techniques (ART)

Your child sex is:

Male (the completion of the questionnaire is finished)

Female (fill in the next section)

E. Has your daughter ever had:

# Y/N

1. Excessive bodily or facial hair (hirsutism)
2. Severe acne
3. Irregular menstrual cycle
4. Unusually painful periods
5. Excessive menstrual bleeding

**Table S1 - Familiar, maternal and paternal steroidopathies in AG and NPG**

|  | AG | | NPG | | p-value |  |  |
| --- | --- | --- | --- | --- | --- | --- | --- |
|  | Present/Total  Valid Answer  (n=47) | % | Present/Total  Valid Answer  (n=45) | % |  | Cramer's V effect |  |
| PCOS | 7 | 14.90 | 6 | 13.30 | 1.0000 | 0.022 |  |
| Endometriosis | 2 | 4.30 | 5 | 11.10 | 0.26197 | 0.129 |  |
| Precocious or delayed puberty | 4 | 8.50 | 2 | 4.40 | 0.6773 | 0.082 |  |
| Breast K | 5 | 10.60 | 8 | 17.80 | 0.3803 | 0.102 |  |
| Ovarian K | 1 | 2.10 | 2 | 4.40 | 0.6126 | 0.065 |  |
| Uterine K | 4 | 8.50 | 3 | 6.70 | 1.0000 | 0,034 |  |
| Prostate K | 1 | 2.10 | 2 | 4.40 | 0.6126 | 0.194 |  |
| Diabetes | 18 | 38.30 | 24 | 53.30 | 0.1500 | 0.151 |  |
| Obesity | 6 | 12.80 | 11 | 24.40 | 0.1513 | 0.15 |  |
| Thyroid pathologies | 27 | 57.40 | 14 | 31.10 | 0.0115 | 0.265 |  |
| Other hormonal/ gynecological conditions | 1 | 2.10 | 2 | 4.40 | 0.6126 | 0.065 |  |
| Maternal Hormonal Treatments | 20 | 42.60 | 19 | 42.20 | 0.9042 | 0.003 |  |
| Gestational diabetes | 1 | 2.10 | 1 | 2.20 | 1.0000 | 0.003 |  |
| Preeclampsia | 2 | 4.30 | 2 | 4.40 | 1.0000 | 0.004 |  |
| Maternal Thyroid pathologies | 7 | 14.90 | 3 | 6.70 | 0.3167 | 0.132 |  |
| Threatened miscarriage | 17 | 36.20 | 12 | 26.70 | 0.2949 | 0.102 |  |
| Stress | 10 | 21.30 | 7 | 15.60 | 0.4518 | 0.073 |  |
| Smoke | 1 | 2.10 | 4 | 8.90 | 0.1984 | 0.142 |  |
| Medications | 17 | 36.10 | 8 | 17.70 | 0.0470 | 0.207 |  |
| ART | 1 | 2.10 | 2 | 4.40 | 0.6121 | 0.065 |  |
| Delivery | Present/Total  Valid Answer  (n=47) | % | Present/Total  Valid Answer  (n=43) | % | p-value |  |  |
| Natural spontaneous eutocic | 19 | 40.40 | 24 | 55.80 | 0.2048 | 0.129 |  |
| Natural induced (oxytocin), dystocic | 5 | 10.60 | 4 | 9.30 | 1.0000 | 0.029 |  |
| Scheduled CS | 12 | 25.50 | 9 | 20.90 | 0.6282 | 0.066 |  |
| Emergency CS | 11 | 23.40 | 6 | 14.00 | 0.2911 | 0.129 |  |
|  | median | IQR | median | IQR | p-value | Hedge' s effect size | Test statistic  Z |
| Week of delivery | 38 | 38.00 to  39.00 | 38 | 38.00 to  40.00 | 0.7897 | 0.028 | 0.267 |
| Mother age | 32 | 29.75 to  36.25 | 32 | 29.00 to  34.50 | 0.1632 | 0.151 | 1.394 |
| Father age | 35 | 30.00 to  39.75 | 35 | 31.00 to  38.00 | 0.6129 | 0.054 | 0.506 |
| Months of breastfeeding | 4 | 1.00 to  9.00 | 2.50 | 0.50 to  7.00 | 0.3744 | 0.095 | 0.888 |

*AG: Autism Group; NPG: NeuroPsychiatric Group; IQR: InterQuartile Range; PCOS: Polycystic Ovary Syndrome; K: Cancer; ART: Assisted Reproductive Technology; CS: Cesarean Section*

**Table S2 -Results of the univariable logistic regression analysis, and corresponding 95% confidence interval**

| *Familiar, maternal and paternal steroidopathies in AG and NPG* | | | |  |
| --- | --- | --- | --- | --- |
| Variable *(Y vs N)* | OR | 95% CI of OR | | p-value |
| PCOS | 1.14 | 0.35 | 3.69 | 0.8303 |
| Endometriosis | 0.36 | 0.06 | 1.93 | 0.2316 |
| Precocious or delayed puberty | 2.00 | 0.35 | 11.50 | 0.4374 |
| Breast K | 0.55 | 0.17 | 1.83 | 0.3304 |
| Ovarian K | 0.47 | 0.04 | 5.34 | 0.5407 |
| Uterine K | 1.27 | 0.27 | 6.03 | 0.7625 |
| Prostate K | 0.47 | 0.04 | 5.34 | 0.5407 |
| Diabetes | 0.54 | 0.24 | 1.24 | 0.1494 |
| Obesity | 0.45 | 0.15 | 1.35 | 0.1551 |
| Thyroid pathologies | 2.98 | 1.27 | 7.035 | 0.0122 |
| Other hormonal/ gynecological conditions | 0.47 | 0.04 | 5.34 | 0.5407 |
| Maternal Hormonal Treatments | 1.05 | 0.46 | 2.41 | 0.9037 |
| Gestational diabetes | 0.98 | 0.06 | 16.12 | 0.9875 |
| Preeclampsia | 0.98 | 0.13 | 7.25 | 0.9821 |
| Maternal Thyroid pathologies | 2.51 | 0.61 | 10.40 | 0.2039 |
| Threatened miscarriage | 1.61 | 0.66 | 3.93 | 0.2939 |
| Stress | 1.51 | 0.52 | 4.39 | 0.4510 |
| Smoke | 0.22 | 0.02 | 2.07 | 0.1873 |
| Medication during pregnancy | 3.56 | 1.25 | 10.11 | 0.017 |
| ART | 0.46 | 0.04 | 5.23 | 0.5292 |
| Induced delivery vs Spontaneous | 1.58 | 0.37 | 6.70 | 0.4965 |
| Scheduled CS vs Spontaneous | 1.68 | 0.59 | 4.83 |  |
| Emergency CS vs Spontaneous | 2.32 | 0.72 | 7.41 |  |
| Variable *(Y vs N)* | OR | 95% CI of OR | | p-value |
| Hirsutism | 2.26 | 0.57 | 9.02 | 0.2463 |
| Severe Acne | 1.10 | 0.24 | 4.99 | 0.9016 |
| Irregular menstrual cycle | 1.11 | 0.30 | 4.07 | 0.8736 |
| Painful menstrual cycle | 0.83 | 0.16 | 4.15 | 0.8154 |
| Excessive bleeding during menstrual cycle | 1.71 | 0.26 | 11.26 | 0.5747 |
| *Hormonal and Metabolic Blood values* | | | |  |
| Variable *(*for each 1 unit increase) | OR | 95% CI of OR | | p-value |
| Estradiol | 1.002 | 0.998 | 1.007 | 0.2742 |
| Prolactin | 0.99 | 0.95 | 1.03 | 0.6957 |
| Testosterone | 1.24 | 1.03 | 1.49 | 0.0209 |
| Folates | 1.07 | 0.94 | 1.23 | 0.2954 |
| Insulin | 1.01 | 0.98 | 1.04 | 0.3402 |
| Cortisol | 1.002 | 0.99 | 1.01 | 0.7157 |
| Vitamine D | 0.99 | 0.96 | 1.03 | 0.8619 |

*OR: Odds Ratio; CI: Confidence Interval; PCOS: Polycystic Ovary Syndrome; K: Cancer; ART: Assisted Reproductive Technology; CS: Cesarean Section; AG: Autism Group; NPG: NeuroPsychiatric Group; Y: Yes; N: No*

**Table S3 - Spearman correlation coefficients to describe relationship between AQ, EQ and SQ scores with Hormonal and metabolic values, and correspondent p-values for statistical significance**

|  |  |  | AG | |  |  | NPG | |  |
| --- | --- | --- | --- | --- | --- | --- | --- | --- | --- |
|  |  |  | F |  | M |  | F |  | M |
|  |  | r | p-value | r | p-value | r | p-value | r | p-value |
| AQ | Testosterone | -0.5 | 0.0512 | -0.7 | 0.0031 | -0.3 | 0.1339 | -0.5 | 0.0591 |
|  | Estradiol | -0.3 | 0.2646 | -0.4 | 0.1119 | 0.02 | 0.9331 | -0.6 | 0.0217 |
|  | Prolactin | -0.2 | 0.5060 | -0.6 | 0.0180 | -0.05 | 0.8370 | -0.5 | 0.0858 |
|  | Cortisol | -0.4 | 0.1639 | 0.02 | 0.9345 | -0.2 | 0.4078 | -0.2 | 0.5905 |
|  | Insulin | -0.002 | 0.9949 | -0.02 | 0.9495 | 0.2 | 0.455 | -0.05 | 0.8725 |
|  | Folates | -0.2 | 0.4015 | -0.1 | 0.7510 | -0.3 | 0.2313 | 0.4 | 0.1591 |
|  | Vitamin D | -0.1 | 0.6255 | 0.5 | 0.0860 | -0.2 | 0.2890 | 0.4 | 0.1796 |
| EQ | Testosterone | 0.2 | 0.3964 | 0.3 | 0.3380 | 0.3 | 0.1439 | 0.2 | 0.5377 |
|  | Estradiol | 0.1 | 0.7753 | 0.3 | 0.3394 | 0.4 | 0.0900 | 0.4 | 0.1830 |
|  | Prolactin | 0.3 | 0.1951 | 0.2 | 0.5658 | 0.03 | 0.9045 | 0.7 | 0.0027 |
|  | Cortisol | 0.4 | 0.0730 | -0.3 | 0.2820 | -0.038 | 0.8721 | 0.8 | 0.0011 |
|  | Insulin | -0.2 | 0.5144 | -0.1 | 0.7849 | -0.3 | 0.1932 | 0.02 | 0.9430 |
|  | Folates | 0.3 | 0.2546 | 0.2 | 0.5245 | -0.05 | 0.8275 | -0.3 | 0.2738 |
|  | Vitamin D | -0.2 | 0.4760 | -0.3 | 0.3190 | 0.3 | 0.2003 | -0.01 | 0.9821 |
| SQ | Testosterone | 0.1 | 0.6637 | 0.7 | 0.0019 | -0.01 | 0.9635 | 0.7 | 0.0123 |
|  | Estradiol | 0.1 | 0.6311 | 0.3 | 0.2866 | -0.2 | 0.4400 | 0.8 | 0.0018 |
|  | Prolactin | 0.1 | 0.8077 | 0.4 | 0.1075 | -0.4 | 0.1193 | 0.3 | 0.2835 |
|  | Cortisol | -0.3 | 0.2453 | -0.1 | 0.6845 | 0.04 | 0.8685 | 0.3 | 0.2749 |
|  | Insulin | 0.1 | 0.7474 | 0.2 | 0.5022 | -0.1 | 0.5600 | -0.01 | 0.9787 |
|  | Folates | 0.3 | 0.3946 | 0.3 | 0.2143 | -0.1 | 0.5398 | -0.5 | 0.1174 |
|  | Vitamin D | 0.04 | 0.8898 | -0.4 | 0.1440 | -0.1 | 0.5666 | -0.1 | 0.6499 |

*AG: Autism Group; NPG: NeuroPsychiatric Group; r: Spearman's rank correlation coefficient; AQ: Autistic Quotient; EQ: Empathizing Quotient; SQ: Systematizing Quotient*
